# A Systematic Review of Analytical Methods for the Separation of Nicotine Enantiomers and Evaluation of Nicotine Sources

**DOI:** 10.1021/acs.chemrestox.2c00310

**Published:** 2023-03-10

**Authors:** Sally Salam, Fatima El-Hajj Moussa, Rachel El-Hage, Ahmad El-Hellani, Najat Aoun Saliba

**Affiliations:** † Department of Chemistry, Faculty of Arts and Sciences, American University of Beirut, Beirut 1107 2020, Lebanon; ‡ Center for the Study of Tobacco Products, Virginia Commonwealth University, Richmond, Virginia 23220, United States; § Division of Environmental Health Sciences, College of Public Health, The Ohio State University, Columbus, Ohio 43210, United States; ⊥ Center for Tobacco Research, The Ohio State University Comprehensive Cancer Center, Columbus, Ohio 43214, United States

## Abstract

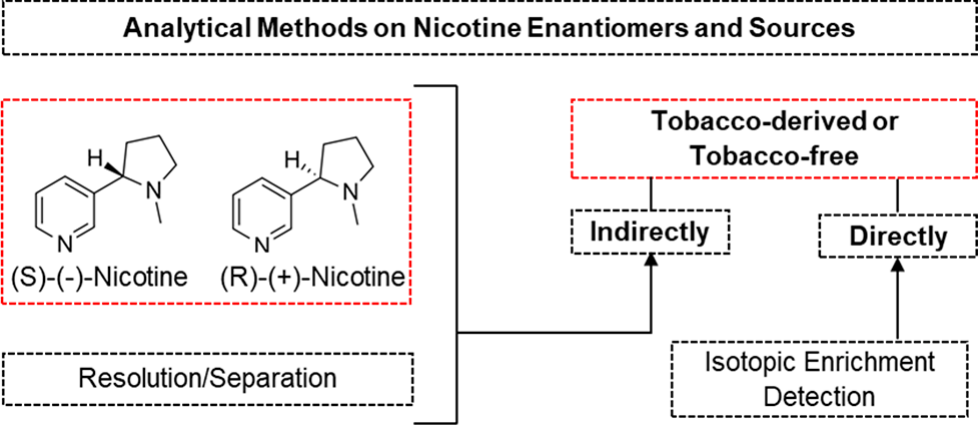

The introduction of synthetic nicotine by the tobacco industry, also promoted as tobacco-free nicotine, presented new challenges for analytical chemists working in tobacco regulatory science to develop and optimize new methods to assess new nicotine parameters, namely enantiomer ratio and source. We conducted a systematic literature review of the available analytical methods to detect the nicotine enantiomer ratio and the source of nicotine using PubMed and Web of Science databases. Methods to detect nicotine enantiomers included polarimetry, nuclear magnetic resonance, and gas and liquid chromatography. We also covered methods developed to detect the source of nicotine either indirectly via determining the nicotine enantiomer ratio or the detection of tobacco-specific impurities or directly using the isotope ratio enrichment analysis by nuclear magnetic resonance (site-specific natural isotope fractionation and site-specific peak intensity ratio) or accelerated mass spectrometry. This review presents an accessible summary of all these analytical methods.

## Introduction

**Figure 1:**
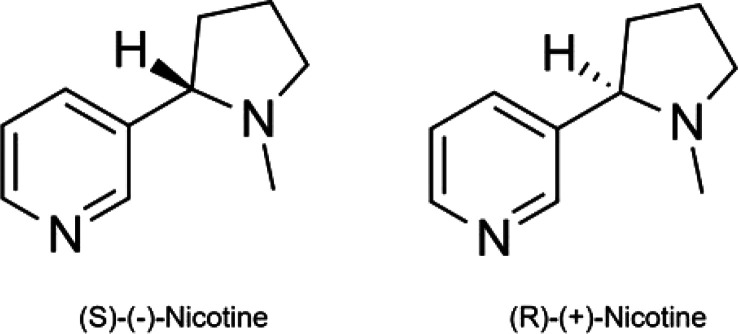
Nicotine enantiomers.

Nicotine is the dominant alkaloid extracted from the *Nicotiana tabacum* plant.^1^ It accounts for ∼95% of all tobacco alkaloids that include nornicotine, anatabine, and anabasine among others.^2^ Nicotine structure is formed of two nitrogen-containing rings, pyridine and pyrrolidine, linked by a carbon–carbon bond. Having a chiral carbon center at the 2′-position of the pyrrolidine moiety (one head of the C–C bond connecting the two rings), nicotine exits in two configurational isomers or enantiomers: (*S*)-(−)-nicotine and (*R*)-(+)-nicotine (Figure 1).^3^ The naturally occurring nicotine or else known as tobacco-derived nicotine (TDN) exists mainly as the (*S*)- enantiomer, while the (*R*)- enantiomer ranges between 0.02 and 0.46% of the total nicotine.^4,5^ On the other hand, nicotine can be synthesized, hence not derived from tobacco, in what is currently marketed as synthetic nicotine or tobacco-free nicotine (TFN).^5,6^ TFN is mostly synthesized as a racemic mixture of (*S*)- and (*R*)-enantiomers (50:50),^7^ which could be enriched to produce ∼99% (*S*)-nicotine, increasing the cost of its synthesis.^8^

Nicotine enantiomers have similar physical and chemical properties, yet in vitro and in vivo studies have shown that they have different pharmacological and toxicological properties.^4,9,10^ For instance, studies have reported that (*S*)-nicotine is more toxic than (*R*)-nicotine in multiple species.^11,12^ Consequently, the racemic mixture of nicotine is more toxic than (*R*)-nicotine.^11^ Moreover, in vivo imaging studies and behavioral and performance tests on animal models have shown that (*S*)-nicotine is significantly more active and pharmacologically potent (up to 9–28 times) than (*R*)-nicotine.^13^ Likewise, in vitro studies on animal cell lines and tissues have reported higher activity on nicotinic receptors of (*S*)-nicotine compared to (*R*)-nicotine.^14^ However, the impact of this stereoselective binding of nicotine and different pharmacological potency on humans has not been tested. Nevertheless, the interest in racemic nicotine mixtures, i.e., TFN, was recently revisited by electronic cigarette (ECIG) manufacturers, like PuffBar and others, to exploit a loophole in the US FDA’s regulatory authority that restricted its oversight of tobacco-derived nicotine.^6,15^ This loophole was recently closed, and the U.S. Food and Drug Administration (FDA) now regulates nicotine from all sources.^16−19^

The uncertainty about TFN’s pharmacological and toxicological effects in humans and the promotion by ECIG manufacturers of TFN as reduced-risk compared to TDN necessitates the development of several analytical methods to resolve/quantify nicotine enantiomers and/or discern the source of nicotine.^20^ For this purpose, several methods were developed using polarimetry, liquid chromatography (LC), gas chromatography (GC), nuclear magnetic resonance (NMR) spectroscopy, and accelerated mass spectrometry (AMS). This systematic review describes the various analytical methods that were reported in the literature to separate and/or quantify nicotine enantiomers and provides an overview of isotopic enrichment methods used to determine nicotine source.

## Methodology

### Search Method

On August 8, 2022, a literature search on PubMed and Web of Science databases with no time restriction was conducted using the following terms: (“Synthetic Nicotine”) OR (“(*R*)-Nicotine”) OR (“(*S*)-Nicotine”) OR (“Racemic Nicotine”) OR (“Nicotine Enantiomers”).

### Inclusion Criteria

Publications were included if the original data focused on the separation of nicotine enantiomers or on the development of analytical techniques to differentiate TFN from TDN.

### Exclusion Criteria

A publication was excluded if it does not report separating nicotine enantiomers or developing analytical techniques that differentiate TFN from TDN. Additionally, non-English, and not peer-reviewed articles were excluded.

### Study Selection and Data Extraction

Two reviewers (SS and FHM) independently examined the title and abstract of each record to evaluate its eligibility. In case of disagreement, a third reviewer (RH) was available to reach a consensus. Records that met the study requirements were then collected for full-text reading and data extraction. All the reviewers met to cross-validate and discuss the extracted data. The data included the techniques used, the scope of application, and outcomes.

## Results

### Included Studies

**Figure 2:**
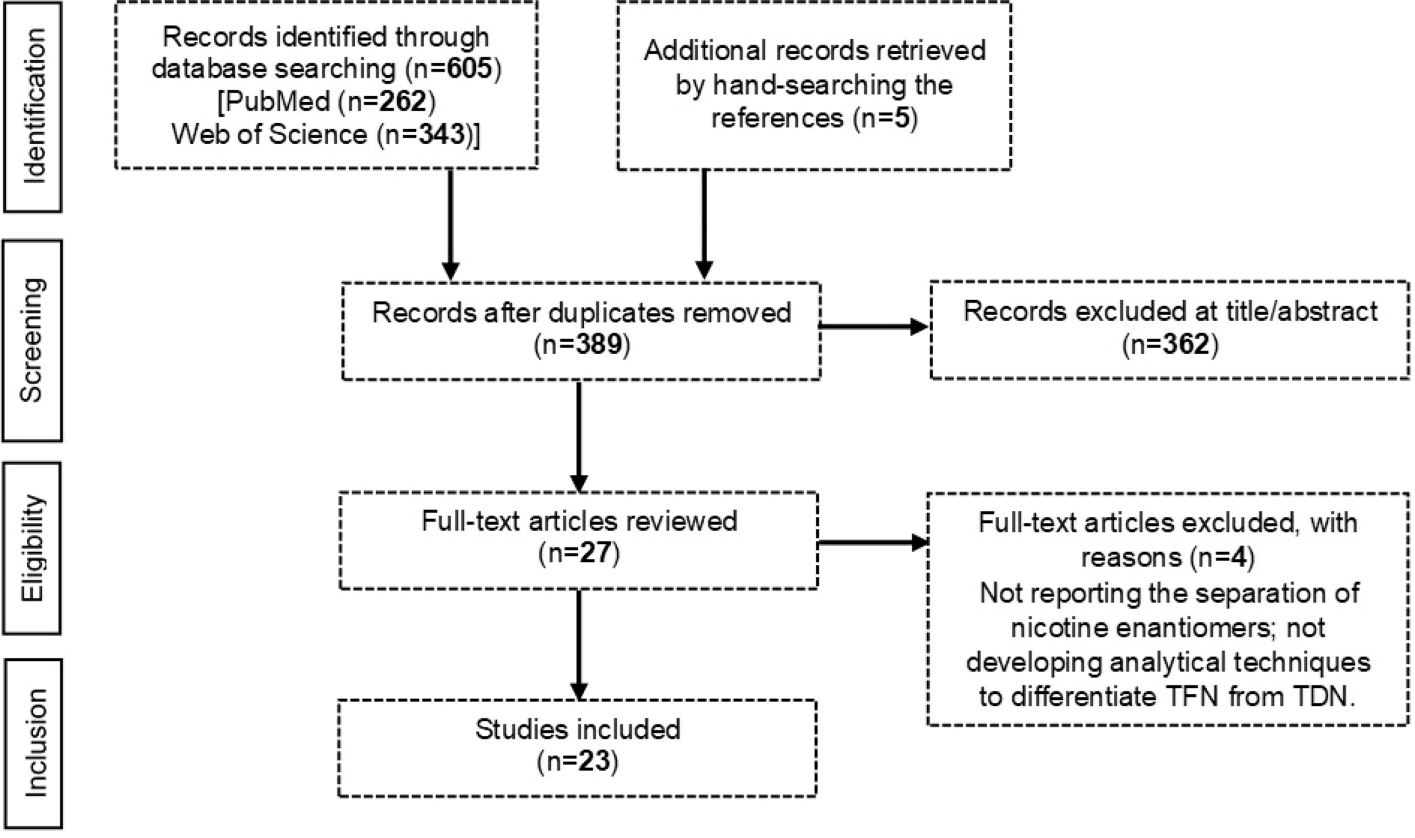
PRISMA diagram summarizing the process of literature selection. Reasons for exclusion of a publication: not reporting the separation of nicotine enantiomers or not developing analytical techniques to differentiate TFN from TDN.

The search yielded 605 records on two databases. An additional 5 references were retrieved by hand-searching the references. After removing duplicates, a total of 389 records were screened by titles and abstracts for inclusion. A total of 362 records were removed at this stage and the full texts of the remaining 27 articles were scanned. Accordingly, a total of 23 articles were included in the review.^1,3−5,8,20−37^Figure 2 shows the PRISMA diagram that summarizes the steps of the selection process.

Results focus on summarizing the analytical methods that were used to separate and quantify nicotine enantiomers and/or to determine nicotine source.

### Analytical Methods

#### Identification and Quantification of Nicotine Enantiomers

Nicotine enantiomers are identified/quantified by specific analytical techniques. Their separation is based on their reaction with a chiral substance such as chiral complexing agents in NMR techniques or on their interaction with a chiral stationary phase in chromatographic techniques. Thus, diastereomers with distinct physical and chemical properties are produced.^38^

##### Polarimetry

The presence of (*R*)-nicotine and (*S*)-nicotine can be identified using the polarimetry technique.^8,23,36^ A sample of (*R*)-nicotine has been used to determine its enantiomeric excess by the polarimetry method.^23^ This method was performed to confirm the results obtained by conducting an ^1^H NMR experiment to determine the enantiomeric ratio of nicotine isomers (vide infra). Although the polarimetry method has not been frequently employed for e-liquid analysis, Duell et al. used this method to report the presence of nicotine enantiomers in Puff Bar e-liquids.^8^ Optically active nicotine enantiomers, (*S*)-nicotine and (*R*)-nicotine, are characterized with specific rotations ([α]_*D*_^20^) of −169° and +169°, respectively. A polarimeter, which measures the optical rotation (α), is then used to find the specific rotation and the enantiomeric excess of the tested samples.^36^ TFN e-liquids of racemic nicotine mixtures are expected to give an α value of 0.0°. On the contrary, slight levorotation was observed (α values below 0.0°) in TFN e-liquid samples. This might be due to the addition of flavors or excess (*S*)-nicotine to the e-liquid. On the other hand, TDN e-liquids, which primarily contain (*S*)-nicotine, would significantly turn the plane of light dextrorotatory in comparison to the TFN e-liquids. In addition to nicotine, e-liquid might contain chiral and optically active flavoring compounds that lead to an alteration of the optical rotation of the mixture. This results in imprecision in finding the ratios of nicotine enantiomers. On another note, depending on the nicotine production pathway, nicotine common impurities (anabasine, nornicotine, cotinine, etc..) may also have chiral centers that will possibly alter the optical rotation, thus affecting the nicotine enantiomeric ratio determination.^8,22^ Consequently, researchers tend to validate the use of polarimetry with NMR spectroscopy or GC-MS methods.^8^

##### Nuclear Magnetic Resonance

NMR spectroscopy is a common technique that has been widely employed to identify and quantify nicotine enantiomers.^8,23,24^ Jaroszewski and Olson demonstrated that ^13^C NMR spectroscopy is a useful method for the assessment of enantiomeric ratios in nicotine samples using a chiral lanthanide complex, tris-[3-(trifluoromethyl-hydroxymethylene)-(+)-camphorato]-ytterbium [Yb(tfc)_3_].^24^ Since proton resonances are broad and their chemical shifts were found to be sensitive to small changes in the ratio between nicotine and [Yb(tfc)_3_], authors reported that ^13^C NMR spectroscopy is more favorable than ^1^H NMR spectroscopy in detecting nicotine enantiomers.^24^ In 1996, Ravard and Crooks described a general method to determine nicotine enantiomeric composition using ^1^H NMR spectroscopy with a different chiral complexing agent: l,l′-binaphthyl-2,2′-diylphosphoric acid.^23^ The ^1^H NMR spectrum of a racemic nicotine sample showed two distinct multiplets attributed to (*R*)-nicotine and (*S*)-nicotine enantiomers. More recently, Duell et al. used ^1^H NMR spectroscopy to determine the ratio of (*R*)-nicotine to (*S*)-nicotine in different Puff Bar e-liquids.^8^ The authors used this method to indirectly evaluate the nicotine source as TFN or TDN. It should be noted that the interaction of nicotine with added chiral complexing agent yields distant peaks in the NMR spectrum.

##### Gas Chromatography

Many GC methods for differentiating nicotine isomers have been reported.^3,8,25,29,32−34^ In 1987, Jacob et al. developed a GC method for determining the enantiomeric purity of nicotine.^25^ This was achieved by demethylating nicotine to give nornicotine that was then converted into the amide derivative of (−)-camphanic chiral acid and then separating the resulting diastereomeric amide derivatives on a capillary GC. Results demonstrated that the enantiomeric purity of (*R*)-nicotine and (*S*)-nicotine was greater than 98%. In 1998, Perfetti and Coleman separated and quantified nicotine enantiomers in a variety of tobacco materials and tobacco smoke using two chiral GC columns (cyclodexB and Rt-BDEX).^33^ Results showed that near baseline resolution was obtained for nicotine enantiomers. The authors reported that 2% is the limit for detecting the (*R*)-isomer in a mixture of (*R*)-nicotine and (*S*)-nicotine. The same technique has also been employed to determine the components of mainstream and sidestream cigarette smoke.^34^ This study demonstrated that the ratio of nicotine enantiomers differed in the mainstream smoke of various cigarettes. In 2007, this method was modified by Liu et al., who used a longer chiral GC column of 60 m in length.^29^ The detection limit of (*R*)-nicotine in a mixture of nicotine enantiomers was considerably improved to reach 0.5%.^29,33^ This led to a better-resolved determination of the ratio of (*R*)-nicotine to total nicotine in cigarette smoke and tobacco samples. In an attempt to assess the possible racemization of (*S*)-nicotine during cigarette smoking simulated using a pyrolysis chamber, Clayton et al. used a chiral GC column to separate nicotine enantiomers in the pyrolysate.^32^ Results showed that there was no rise in (*R*)-nicotine levels over a wide pyrolysis temperature range.

Similar to their NMR assay, Duell et al. used a GC method to differentiate between TDN and TFN e-liquids using a Beta DEX 120 GC column.^8^ In a similar study, e-liquids were tested using a chiral GC column (CHIRALDEX G-TA) but the nicotine isomers retention times were considered excessively long, and quantitative analysis was challenging because the peaks were not entirely separated;^3^ in response, the authors developed an LC method to separate nicotine enantiomers.

##### Liquid Chromatography

Similarly, LC has been extensively employed for nicotine chiral separation.^1,3−5,20,21,26−28,30,35^ In 1987, Armstrong et al. reported the use of a packed LC microcolumn with a mobile phase saturated with a chiral selector (β-cyclodextrin) to separate nicotine enantiomers.^27^ The baseline separation is usually reflected by a resolution factor (R_s_) which is a quantitative measurement of the degree of separation between two chromatography peaks.^39^ If R_s_ is ≥1.5, this reflects a baseline separation of the peaks.^27^ In this study, nicotine enantiomers had a factor of 1.7, indicating that (*R*)-nicotine and (*S*)-nicotine were well resolved.^27^ Compared to traditional LC packed column methods, microcolumn LC offers three additional benefits: more theoretical plates, fewer quantities of the frequently expensive chiral additives for chiral mobile phase work, and fewer volumes of samples/solvents.^27^ However, this was a time-consuming method with a long analysis time (4 h). Later, this same group evaluated the use of a bonded β -cyclodextrin chiral stationary phase in the LC reversed-phase mode for the separation of nicotine enantiomers, but this separation could not be achieved.^26^ Demetriou et al. developed a sensitive and reproducible LC method using a commercially available chiral α_1_-acid glycoprotein stationary phase and a binary solvent program consisting of dipotassium phosphate and decanoic acid and methanol.^35^ Armstrong et al. analyzed nicotine enantiomeric composition in a variety of consumer products, natural products, and commercial reagents.^1^ The selected column for the LC method was the Chiralcel OJ column with a stationary phase of silica gel and adsorbed cellulose tris(4-methylbenzoate). Tang and co-workers described an effective and efficient LC method that achieved the optimum separation of nicotine enantiomers in a shorter elution time. However, the elution order was opposite to that previously reported by Demetriou et al.^30,35^ This method utilized two derivatized cellulose chiral stationary phases (tris(4-methylbenzoyl) cellulose) and tris(3,5-dimethylphenyl carbamoyl) cellulose) operating in normal phase to separate (*R*)- and (*S*)-nicotine. This study suggested that the choice of chiral stationary phase, chiral selectors, and mobile phase composition can significantly affect enantio-resolution and solute retention. The use of chiral selectors or columns relies on the differential spatial interaction of the two nicotine isomers with these molecules in the mobile or stationary phase.

In 2018, Hellinghausen et al. accomplished a rapid enantiosepartion of nicotine in less than 20 s (R_s_ = 2.6) using an optimized ultrafast LC technique.^21^ A modified macrocyclic glycopeptide stationary phase was used with a mobile phase composed of methanol and ammonium formate. Moreover, a novel, rapid, and sensitive ultraperformance LC-MS/MS has been developed in 2019 by Ji and co-workers.^4^ After optimization, the selected column was Chiralpak AGP, the mobile phase consisted of ammonium formate with ammonium hydroxide and methanol in an isocratic elution program. Nicotine enantiomers in tobacco leaves and different tobacco products were successfully resolved within 10 min. The same group reported a rapid and effective LC-MS method for determining the concentration of nicotine enantiomers in TFN products using an LC equipped with a triple quadrupole MS.^20^ The stationary phase was made of a modified macrocyclic glycopeptide bonded to superficially porous particles. An isocratic elution was carried out using a mobile phase of methanol and ammonium trifluoroacetate and the results demonstrated a very short retention time (less than 2 min) with R_s_ = 3.0. In this study, the differences between various TDN and TFN products were highlighted. All studied TFN products contained a racemic nicotine mixture; however, only small quantities of (*R*)-nicotine were detected in TDN products. Similarly, a normal phase LC procedure was developed by Zhang et al. to effectively separate (*R*)-nicotine and (*S*)-nicotine in different TFN and TDN samples, and indirectly determine the nicotine source.^5^ Small quantities of (*R*)-nicotine ranging from 0.02% to 0.76% were measured in the majority of the tested samples much lower than that in TFN e-liquid and standard racemic nicotine mixture (50%). They concluded that if the (*R*)-nicotine ratio is around 1%, nicotine is expected to be naturally derived from tobacco leaves, however, if this ratio is 50% or 100%, nicotine is assumed to be synthesized. Also, an LC coupled with a diode array detector was utilized for a complete separation of nicotine enantiomers in various e-liquids: natural, and TFN. Only in TFN e-liquids, both enantiomers were detected in similar proportions.^3^ Moldoveanu recently developed a method for the analysis of nicotine enantiomers using LC-MS/MS from seven different commercial sources.^28^ The Chiracel OJ-3 column was used to perform the LC separation in isocratic conditions. The author detected a small percentage (0.21–2.19%) of (*R*)-nicotine in commercially available nicotine obtained from tobacco which varies depending on the source of the tobacco and perhaps the extraction method. An e-liquid advertised as containing synthetic (*S*)-nicotine was found to contain a very low percentage of (*R*)-nicotine whereas a racemic nicotine mixture standard was shown to contain 50/50 (R)/(*S*)-nicotine.

#### Identification of Nicotine Source

##### Indirect Identification of Nicotine Source

As shown in the previous section, several analytical methods have been used to separate and quantify (*R*)-nicotine and (*S*)-nicotine in a wide variety of matrices. The ratio of (*R*)-nicotine present in the sample helps in assigning the nicotine source. However, if synthetic nicotine was purified to become 99% (*S*)-nicotine, TFN becomes indistinguishable from TDN if the previously described analytical methods were used. Other analytical methods were found to be used for this purpose.^22^ In fact, it was suggested that TFN and TDN could be distinguished by detecting specific impurities like tobacco-specific nitrosamines, nicotine degradants, and metals in TDN samples from one hand and synthetic precursors and residual solvent impurities in TFN samples from the other.^22^ However, this approach is challenged by enhanced purity in processing TFN and TDN products and also by the detection limits of the adopted analytical methods.

##### Direct Identification of Nicotine Source

*Nuclear Magnetic Resonance*. In 1981, Martin and Martin introduced the site-specific natural isotope fractionation determined by NMR (SNIF-NMR) that gives site-specific isotope ratios of ^2^H/^1^H (deuterium/proton).^40^ This technique was later used by the same group and others to authenticate the source compounds including vanillin, benzaldehyde, and sugars in juices and wines among other matrices.^41−45^ Martin and co-workers used this method to analyze nicotine from different regions of the world.^37^ However, this method was recently criticized because it needs either a high quantity of the sample or a prolonged runtime, and also it should be coupled with isotope ratio mass spectrometry to determine the overall ^2^H/^1^H isotopic ratio of nicotine. Alternatively, a site-specific peak intensity ratio NMR (SPIR-NMR) was introduced to analyze the nicotine source by directly comparing ^2^H/^1^H SPIR values derived from ^1^H and ^2^H NMR spectra.^31^ This technique was used to differentiate TFN from TDN in natural nicotine samples and synthetic (*R*)-nicotine and racemic nicotine but not in tobacco products.

*Accelerator Mass Spectrometry*. Cheetham and co-workers explored different techniques to distinguish between TDN and TFN including screening for impurities (nicotine degradants and metals), chiral separation of nicotine and nornicotine enantiomers, and radiocarbon analysis.^22^ However, only radiocarbon analysis of ^14^C successfully differentiated TDN from TFN in all tested samples. The standard procedure ASTM D6866 which uses AMS to separate ^14^C from the other two carbon isotopes (^12^C and ^13^C) is the most frequently employed method to determine the radiocarbon content.^46^ Measuring the ^14^C content of a sample indicates if the material is synthesized, biologically derived, or a combination of both. Usually, the outcome is expressed as% Biocarbon. Purely synthetic substances derived from petrochemicals will yield a result of 0% Biocarbon, while pure biological substances will give a result of 100% Biocarbon. Depending on the proportions of each source, synthetic materials made from a combination of petrochemical and biological feedstocks will fall between 0 and 100% Biocarbon. As determined in this study, there were three possible outcomes for the radiocarbon results of the nicotine analysis. TFN e-liquids had a% Biocarbon of less than 40% whereas TDN e-liquids had a% Biocarbon of 100%. Accordingly, adulterated e-liquids with TFN being mixed with TDN will fall between the two extremes (this has not been detected in any commercially available tobacco product). Yet, this technique is not selective during separation. It determines the total% Bio-Carbon of the analyzed sample and not that of nicotine alone. Thus, this technique requires nicotine extraction pretreatment.^22^

Table 1 summarizes the characteristics of the reviewed analytical methods for the separation of nicotine enantiomers and the evaluation of nicotine source.

**Table 1:** Characteristics of Analytical Methods Used to Separate and Quantify Nicotine Enantiomers and/or to Determine Nicotine Source

Method	Experimental Details			Year, Ref	Samples
Identification and Quantification of Nicotine Enantiomers					
Polarimetry	Autopol polarimeter at 589 nm			2021, (8)	Determination of nicotine enantiomeric ratio in different Puff Bar e-liquids
Polarimetry	NA			1996, (23)	Determination of the enantiomeric ratio of nicotine in standard nicotine samples
Polarimetry	Static Polarimeter			1991, (36)	Quantitation of the relative enantiomeric purity of nicotine samples
^13^C NMR	Frequency: 100 MHz	Complexing agent: tris-[3-(trifluoromethyl-hydroxymethylene)-(+)-camphorato]-ytterbium		1994, (24)	Determination of enantiomeric purity of nicotine in chewing gums, skin absorption patches, inhalators, and nasal sprays
^1^H NMR	Frequency: 400 MHz				
^1^H NMR	Frequency: 300 MHz	Complexing agent: l,l′-binaphthyl-2,2′-diylphosphoric acid		1996, (23)	Determination of enantiomeric purity of tobacco alkaloids and nicotine-like compounds
^1^H NMR	Frequency: 600 MHz	Complexing agent: l,l′-binaphthyl-2,2′-diylphosphoric acid		2021, (8)	Determination of nicotine enantiomeric ratio in different Puff Bar e-liquids
GC-Nitrogen phosphorus	Column: SE-54 fused silica (25m, 0.2 mm)		Rt ≈ 8 min	1988, (25)	Separation of (−)-camphanic acid amide derivatives of racemic nicotine and (*R*)-nicotine
GC-Mass Selective	Column: CyclodexB and Rt-BDEX (30 m, 0.25 mm, i.d. 0.25 μm)		Rt ≈ 159 min	1998, (33)	Separation and quantification of nicotine enantiomers in extracts of tobacco seeds, processed tobacco suspensions, reconstituted tobacco sheet materials, individual tobacco varieties, blends of tobaccos, and cigarette smoke condensate
				1998, (34)	Analysis of enantiomeric distribution of nicotine in mainstream and sidestream in different cigarettes smoke
GC-Nitrogen phosphorus	Column: DB-CyclodexB (60m, 0.25 mm, i.d. 0.25 μm)		Rt ≈ 149 min	2008, (29)	Determination of the enantiomeric composition of nicotine in racemic standards and 20 Tobacco samples
GC-MS	Column: CyclodexB (30m, 0.25 mm, i.d. 0.25 μm)		Rt ≈ 73 min	2010, (32)	Measurement of the enantiomeric purity of the pyrolysate (−)- (S) nicotine
GC-MS	Column: β-DEX 120 (30m, 0.25 mm, i.d. 0.25 μm)		Rt ≈ 39 min	2021, (8)	Determination of nicotine enantiomeric ratio in different Puff Bar e-liquids
GC-MS	Column: CHIRALDEX G-TA (20m, 0.25 mm, i.d. 0.12 μm)		Rt ≈ 149 min^a^	2021, (3)	Quantitative analysis of nicotine isomers in urine and saliva from e-liquid smokers and in e-liquids sold in South Korea
LC-Variable Wavelength	Column: Packed microcolumn (1m, 250 μm i.d.)	Mobile phase: β-cyclodextrin saturated in acetonitrile–water (20:80)	Rt ≈ 4 h	1987, (27)	Enantiomeric separation of racemic nicotine and other related compounds
HPLC-Variable Wavelength	Column: β-cyclodextrin (25, 0.46 cm)	Mobile phase: Acetonitrile/aqueous triethylammonium acetate	a	1988, (26)	Enantiomeric resolution of nicotine samples obtained by racemization or complete synthesis
HPLC-Diode array	Column: Chiral α1-acid glycoprotein (100, 4.0 mm i.d., 5 μm)	Mobile phase: Binary program (Dipotassium phosphate and decanoic acid) and (methanol)	Rt ≈ 4 min	1993, (35)	Separation and quantification of nicotine enantiomers in nicotine standard
HPLC-UV	Column: Chiralcel OJ (25, 0.46 cm i.d.)	Mobile phase: Hexane:ethanol:trifluoroacetic acid:trimethylamine (85:15:0.075:0.0375) v%	Rt ≈ 15 min	1998, (1)	Determination of the enantiomeric composition of nicotine in smokeless tobaccos, strains of tobacco leaf, pharmaceutical products, and commercial reagents
HPLC-Diode array	Column: Chiralcel OJ (25 cm, 4.6 mm i.d.)	Mobile phase: Hexane:methanol:trifluoroacetic acid (95:4.98:0.02) v%	Rt ≈ 25 min	1998, (30)	Separation of nicotine enantiomers in nicotine standard
HPLC-Diode array	Column: NicoShell (50 × 4.6 mm^2^ i.d.)	Mobile phase: Methanol:ammonium formate (100:0.2) wt%	Rt ≈ 18 s	2018, (21)	Enantiomeric separation of nicotine-related compounds in different racemic standards
LC-MS/MS	Column: Chiralpak AGP (150 × 4 mm^2^ i.d)	Mobile phase: Ammonium formate with 0.3% ammonium hydroxide and methanol (90:10) v%	Rt ≈ 10 min	2019, (4)	Determination of tobacco alkaloid enantiomers in tobacco and wide range of tobacco products
HPLC-Diode array and LC-MS	Column: Macrocyclic glycopeptide chiral stationary phase (100 × 4.6 mm^2^ i.d.)	Mobile phase: Methanol:ammonium trifluoroacetate (100:0.1) wt%	Rt ≈ 2 min	2017, (20)	Evaluation of enantiomeric ratio of nicotine in commercial tobacco products and TFN products
LC-MS/MS	Column: Chiracel OJ-3 (250 × 4.6 mm^2^)	Mobile phase: Hexane: Ethanol (85:15) v%	Rt ≈ 8 min	2022, (28)	Evaluation of enantiomeric ratio of nicotine in nicotine pyrolyzates at different temperatures and exposure time, different types of tobacco, smoke from combustible cigarettes and heated tobacco products, e-liquids, and particulate matter from ECIG aerosol
HPLC	Column: Chiralcel OD-H (25 × 0.46 cm^2^)	Mobile phase: Hexane: Methanol (98:2) v%	Rt ≈ 9 min	2018, (5)	Evaluation of (*S*)-(−)-nicotine and (*R*)-(+)-nicotine in tobacco leaf, cigarette, smokeless tobacco, and e-liquid samples
HPLC-UV	Column: Chiral OD-H column (4.6 × 250 mm, 5 μm)	Mobile phase: Gradient elution using *n*-hexane and ethanol	Rt ≈ 7 min	2021, (3)	Quantitative analysis of nicotine isomers in urine and saliva from e-liquid smokers and in e-liquids sold in South Korea
Identification of the Nicotine Source					
^2^H NMR SNIF	Frequency: 76.7 MHz			1997, (37)	Analysis of nicotine from tobacco leaves from different regions of the world.
^1^H NMR-SPIR	Frequency: 800 MHz			2019, (31)	Distinction between natural and synthetic nicotine samples
AMS	Standard procedure ASTM D6866 that separates ^14^C from the other isotopes			2022, (22)	Differentiation between TDN and TFN in e-liquids from different companies

a No complete separation, NA: not available.

## Discussion and Conclusion

Analytical methods for the enantioseparation of nicotine have been developed through the years. By scoping the GC methods included in this review, some studies were outdated due to long run time (up to 4 h).^3,27,29,33,34^ Additionally, two references reported no complete separation.^3,26^ The polarimetry method was shown to be among the easiest methods, yet the data analysis could be complicated by the presence of other chiral compounds such as flavors in the matrix. A common practice among the different research groups is to combine polarimetry with other methods to cross-validate the results. The NMR detection of (*R*)-nicotine and (*S*)-nicotine is an easy method to adopt, but it needs access to an NMR instrument and the sample should not have any considerable impurities in the low field of the NMR spectrum (region 8–8.5 ppm). On the other hand, chromatography methods (i.e., GC and LC) allow for simultaneous purification of the sample and enantiomeric separation of nicotine.^47^ Nonetheless, the reviewed analytical methods that determine the (*R*)-/(*S*)-nicotine ratio can benefit tobacco regulation if the use of TFN in tobacco products prevails. This can be also used to study the abuse liability of TFN tobacco products.

As mentioned before, TDN exists predominately in the (*S*)-enantiomer, containing only minor amounts of the (*R*)-enantiomer. Thus, if the percentage of (*R*)-nicotine that is detected using the aforementioned analytical techniques exceeds a certain percentage (>1.5%), this indicates that the tested samples could contain TFN.^22^ Moreover, the “enantiomeric ratio” and the “impurities” approaches will not be able to account for the presence of TFN extra-purified samples that are made only of the (*S*)-enantiomer. Accordingly, it is suggested that SNIF-NMR, SPIR-NMR, or AMS methods are used to confirm the nicotine source.^22,31,37^

## Abbreviations

TDN: tobacco-derived nicotine
TFN: tobacco-free nicotine
ECIG: electronic cigarette
LC: liquid chromatography
GC: gas chromatography
NMR: nuclear magnetic resonance
AMS: accelerated mass spectrometry
SNIF: site-specific natural isotope fractionation
SPIR: site-specific peak intensity ratio
